# Effects of photobleaching on selected advanced glycation end products in the human lens

**DOI:** 10.1186/s13104-015-0977-3

**Published:** 2015-01-16

**Authors:** Thomas Holm, Cibin T Raghavan, Rooban Nahomi, Ram H Nagaraj, Line Kessel

**Affiliations:** Department of Ophthalmology, Glostrup Hospital, Ringvej 57, 2600 Glostrup, Denmark; Department of Ophthalmology & Visual Sciences, Case Western Reserve University School of Medicine, Cleveland, Ohio USA; Present address: Department of Ophthalmology, University of Colorado School of Medicine, 12800 East 19th Avenue, RC-1 North 5102, Aurora, CO 80045 USA

**Keywords:** Advanced glycation end products, Cataract, Laser, Treatment

## Abstract

**Background:**

Cataract is the leading cause of blindness, especially in the developing world. To ease access to treatment, we have proposed that cataract could be treated non-invasively by photobleaching of the chemically modified proteins responsible for cataract formation. The present study was aimed at examining the optical and biochemical effects of the proposed treatment.

**Methods:**

Human donor lenses were photobleaced using a 445 nm cw laser. Lens optical quality was assessed before and after photobleaching by light transmission and scattering. The concentration of the advanced glycation end products (AGEs) pentosidine, argpyrimidine, carboxymethyllysine, hydroimidazolone was measured.

**Results:**

Transmission increased and AGE-related fluorescence decreased significantly after photobleaching but no changes were observed in the concentration of the measured AGEs.

**Conclusions:**

We found a significant effect of the photobleaching treatment on lens optical parameters but we could not associate the optical findings to a change in the concentration of the AGEs we measured. This finding suggests that other AGEs were responsible for the observed photobleaching of the human lens after laser treatment. The biochemical nature of the photochemical reactions associated with photobleaching remains to be elucidated.

## Background

In spite of an effective and relatively safe treatment, cataract remains the leading cause of blindness worldwide accounting for nearly 20 million cases [1]. The majority of blind from cataract live in the developing nations that have poor coverage of ophthalmic care and inadequate access to cataract surgery [2]. At present, cataract development cannot be prevented so the only way to combat blindness from cataract is by making cataract treatment more easily accessible. In the Western world, blindness from cataract is rare [3] but cataract remains an important cause for impaired visual function [4]. In US, cataract extraction and IOL implantation is the most frequently performed procedure and the single largest item in the Medicare budget. In the Western world the need for cataract treatment is estimated to double within the next two decades [5] due to increased number of aged individuals in combination with a trend towards patients demanding treatment at an earlier stage in the disease process [6].

During aging and cataract formation the optical quality of the lens deteriorates because of increased scattering and absorption of incident light. Lens transparency is intimately related to the three-dimensional arrangement and optical interaction of the intrinsic lens proteins [7,8]. Cataract is a protein conformational disorder [9] whereby a number of posttranslational protein modifications induce a conformational change in the intrinsic lens proteins and/or crosslinking of lens proteins leading to a disorder in the arrangement of proteins. Furthermore, some of the protein modifications are chromophores that directly absorb light. The end result is decreased transmission of light to the retina and hence an impaired vision.

The posttranslational protein modifications involved in lens ageing have not been fully elucidated yet but they include deamidation [10], non-enzymatic cleavage [11], truncation [12] and racemization [13] and non-enzymatic glycation leading to the formation of advanced glycation end products [14]. Advanced glycation products include chromophores and fluorophores, some of which are formed as protein crosslinking adducts. Numerous advanced glycation products have been detected in the human lens and their levels have been found to increase with age and increased further in cataractous lenses [14]. In theory, manipulating some of the protein changes causing optical disturbance in cataract could provide an interesting basis for a non-surgical treatment of cataract. It has been shown by us [15,16] and others [17] that some of the age-related optical changes in the human lens can be reversed by non-invasive irradiation with short-wavelength or infrared laser irradiation. The aim of the present study was to examine if the optical changes observed in photobleached human lenses are caused by a change in the concentration of advanced glycation end products.

## Methods

Intact human lenses were obtained from the Cornea Bank, Amsterdam (Euro Tissue Bank, Beverwijk, The Netherlands). Lenses were shipped and stored in Castor Oil (Sigma Aldrich, Brøndby, Denmark) as previously described [18]. Lenses maintained optical clarity for several weeks in castor oil. The study adhered to the tenets of the Helsinki Declaration. The study was approved by the Regional Ethical Committee of the Capitol Region of Denmark (H-3-2011-035).

### Optical analyses

Lenses were placed in 5 mm path length quartz cuvettes filled with castor oil for optical analyses (transmission and scatter). One lens from a pair was kept as control and the other was photobleached with a 445 nm cw laser. Optical performance of the lens was measured before and after photobleaching.

White light transmission spectra were recorded as described previously [19]. In short, a fibre-coupled broad-band white light source (DT-Mini-2-GS, Micropack, Ocean Optics, the Netherlands) was placed close to the anterior lens surface and transmitted light was collected close to the posterior lens surface by an integrating sphere (FOIS-1, Ocean Optics, the Netherlands) that was coupled to a spectrometer using an optical fibre (USB4000, Ocean Optics, The Netherlands). Transmission was measured through the central 2 mm of the lens in an anterior-posterior direction. Transmission was calculated as the ratio between transmitted and incident light after correction for background light levels.

Forward scattered light was measured as the angular distribution of a 661 nm laser (Coherent Cube, 661 nm, 100 mW, diode cw laser, spotsize 1 mm, divergence 1 mrad, Coherent Inc., Santa Clara, CA, USA). The laser light was aimed at the center of the lens in anterior-posterior sagittal direction. The intensity of the transmitted laser light was measured at different angles in 0.1 degree steps using a fibre coupled spectrometer (USB4000, Ocean Optics, The Netherlands). Scattering was evaluated as the angular intensity profile of the forward scattered light.

### Photobleaching

Lenses were photobleached in a 4x10 mm area covering the central part of the lens using a 445 nm laser (500 mW, RGBLase LLC, Fremont, CA, USA). An irradiation dose of 1.5 kJ/cm^2^ was used.

### Lens sample preparation and measurement of advanced glycation end products

A cylindrical sample was taken from the central part of the lens in an anterior-posterior direction using a corkborer with an internal diameter of 3.5 mm. The lens samples were placed in 500 μl lysis buffer (150 mM NaCl, 50 mM Tris–HCl (pH = 7.4), protease inhibitor (Roche 04 693 124 001) and phosphatase inhibitor (Roche 04 906 837 001)). The lenses were mechanically homogenized with an Ultra-Turrax T8 (IKA Labortechnik) and left to lyse for 30 minutes on ice. Samples were lyophilized over night. After lyophilization the lens samples were kept at −80° C until they were analyzed for the concentration of advanced glycation end products.

### Measurement of pentosidine and argpyrimidine

Lyophilised lens samples (5 mg/ml) were hydrolyzed using 6 N HCl, dried and reconstituted in 250 μl of water and filtered through 0.45 μm centrifugal filters. Pentosidine and argpyrimidine were measured by reversed-phase HPLC using a C_18_ column as previously described [20]. The amino acid content of the acid hydrolysates was estimated by the ninhydrin assay [21]. Standard curves for argpyrimidine and pentosidine were generated using respective synthetic standards run under the same conditions [22]. The data were expressed as picomoles of pentosidine or argpyrimidine per μM amino acid.

### Measurement of AGE fluorescence

Lyophilised lens samples (5 mg/ml) were suspended in 50 mM sodium phosphate buffer pH 7.4 and sonicated for 5 min with cooling. A power setting of 4 (approximately 40 W) and a 30% duty cycle were used with Branson Digital Sonifier (model S-450D, Branson Ultrasonics Co., Danbury, CT). Sonicated samples were centrifuged at 21,000 g for 30 min at 4°C. Protein was estimated in the supernatant using the BCA method. Fluorescence was measured in a Horiba Jobin Yvon Fluromax-4 Spectrofluorometer at excitation/emission wavelengths of 335/385 nm (pentosidine-like) and 370/440 nm (argpyrimidine-like) and expressed as arbitrary units.

### Direct ELISA for CML and HI

Microplates were coated with 4 μg protein/well and direct ELISA was performed as previously described [23].

## Results

**Figure 1:**
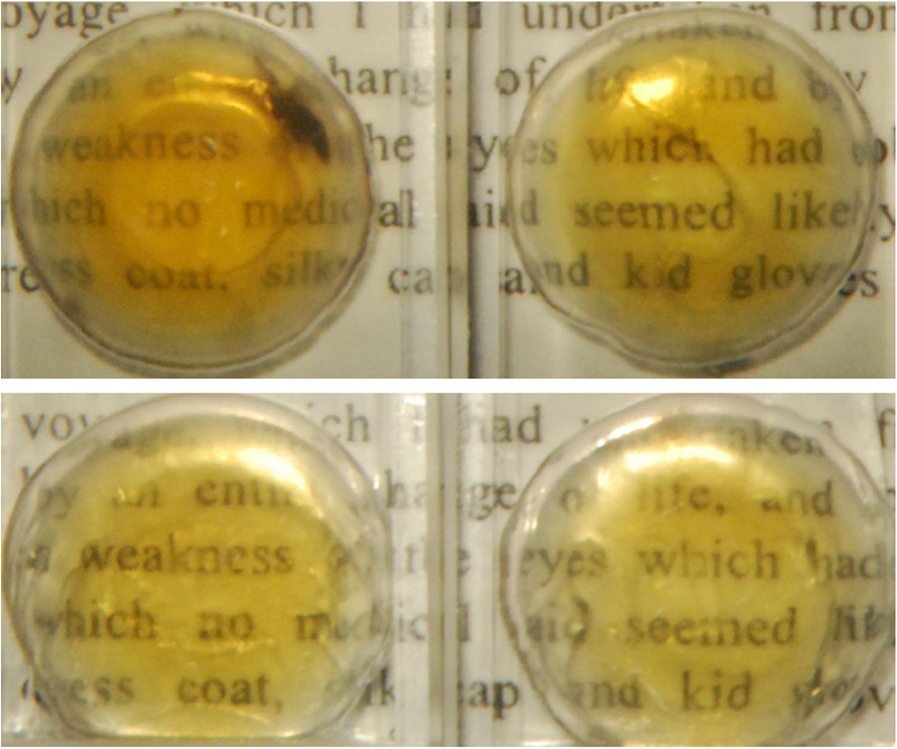
**Photographs of two pairs of human donor lenses aged 74 years (upper panel) and 54 years (lower panel).** The untreated, control lens is shown on the left side of the photograh. The treated lens is shown on the right side of the photograph. The treated lenses were photobleached in a 4x10 mm horisontal area covering the central part of the lens.

Fifteen lens pairs were included in the experiments. Age of the lenses ranged from 42 to 84 years (mean ± SD: 64.1 ± 12.0). One lens from a pair was photobleached and the other lens from the same pair was kept as an untreated control. All lenses were from organ donors and they demonstrated age-related optical changes such as increased yellowing. An example of two photobleached lenses and their untreated counterpart lenses is shown in Figure 1.

### Transmission

**Figure 2:**
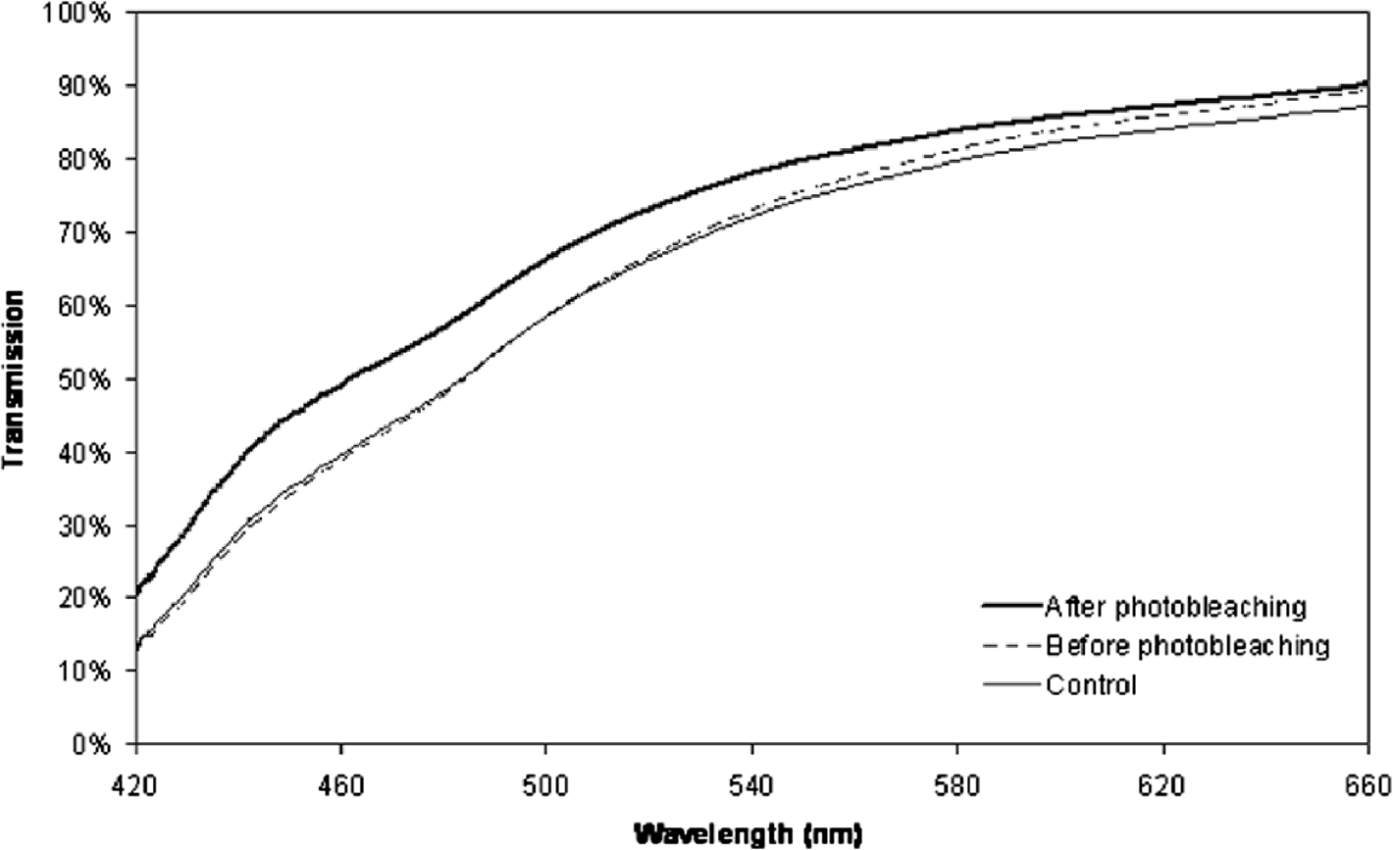
**Transmission characteristics in untreated lenses and in lenses before and after photobleaching.** The graph shows the mean spectral transmission characteristics for all lenses included in the experiments as control lenses (untreated, n = 15) and all the photobleached lenses (n = 15) before and after the photobleaching procedure.

Transmission increased significantly after photobleaching compared to untreated lenses (Table 1 and Figure 2). On average, the transmission of white light (450–650 nm) increased by 6.9% after photobleaching and the transmission of blue light increased by 24.6% when the spectra of the individual lenses were compared. Untreated (control) lenses and the lenses that were photobleached had comparable transmission characteristics before the photobleaching treatment, Table 1. Transmission increased at all wavelengths but most markedly at shorter wavelengths where absorption was most marked prior to photobleaching with a peak increase at 447 nm.

**Table 1:** **Transmission before and after photobleaching**

	***White light transmission 450–650 nm***	***Blue light transmission (450–490 nm)***	***Paired t-test, white light transmission***	***Paired t-test, blue light transmission***
Control lens	0.70 ± 0.11 (0.48-0.84)	0.45 ± 0.15 (0.19-0.67)		
Treated lens, before treatment	0.71 ± 0.09 (0.57-0.85)	0.45 ± 0.14 (0.22-0.67)	0.47	0.62
Treated lens, after treatment	0.76 ± 0.08 (0.64-0.89)	0.54 ± 0.13 (0.33-0.75)	<0.001	<0.001

Values are presented as mean ± SD (range). For the paired t-test, the treated lens before treatment was compared to the control lens whereas the treated lens after treatment was compared to the same lens before treatment.

### Scattering

**Figure 3:**
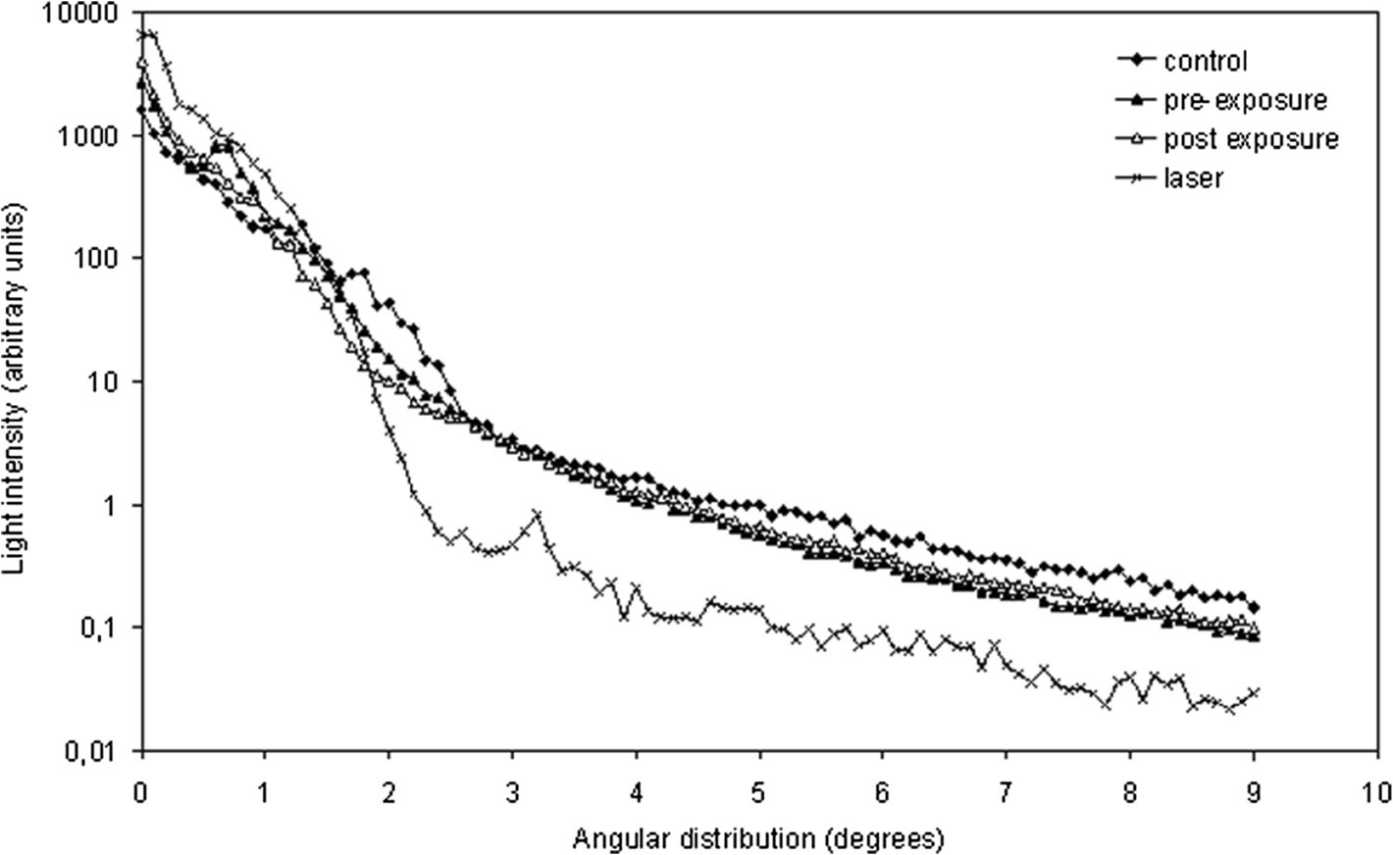
**Scattering was measured as the angular intensity profile of forward scattered light using a red laser light source.** The graph shows the mean of all control lenses and the mean of the lenses before and after photobleaching. The scattering profile of the red laser through a cuvette filled with castor oil is shown for comparison. The scale on the y-axis is logarithmic.

Scattering was measured as the angular distribution of forward scattered red light, see Figure 3. The ratio of the intensity of light (AUC) scattered from 0 to 0.5 degree relative to the intensity of light (AUC) scattered from 0 to 9 degrees was used as a scattering measurement. All lenses showed very little light scattering before treatment and after treatment. There were no age-related changes in scattering (p = 0.66). The percentage of light scattered less than 0.5 degrees was 65.8% ±17.6% (mean ± SD) in control lenses versus 68.3% ± 19.1% in treated lenses before the photobleaching procedure. After photobleaching the percentage of light scattered less than 0.5 degrees had increased to 76.9% ± 10.7% (mean ± SD) but the difference in light scattering before and after photobleaching was not statistically significant (p = 0.16).

### Advanced glycation end products

The concentration of advanced glycation end products was measured by HPLC, ELISA and fluorescence emission. The concentration of argpyrimidine and pentosidine was measured by HPLC and we did not find any difference in the concentration of these advanced glycation end products between the control and the photobleached lenses (Table 2). The concentration of carboxymethyllysine and hydroimidazolone was measured by ELISA and we also did not find any difference in the concentration between control and photobleached lenses (Table 2).

**Table 2:** **Concentration of advanced glycation end products in control lenses and treated lenses**

	Pentosidine** (n = 10)	CML*(n = 8)	HI* (n = 8)	Argpyrimidine** (n = 8)
Control lens	1.10 (0.22)	0.15 (0.04)	0.04 (0.02)	3.76 (3.89)
Treated lens	1.19 (0.46)	0.15 (0.04)	0.04 (0.02)	2.25 (1.53)
Signed rank sum test (p-value)	0.06	0.84	0.83	0.38

Concentration of advanced glycation end products in control lenses and treated lenses. CML: carboxymethyllysine. HI: hydroimidazolone. CML and HI were measured by ELISA and pentosidine and argpyrimidine were measured by fluorescence HPLC . *Absorbance units. **pmol/μmole amino acid. N denotes number of lens pairs.

**Figure 4:**
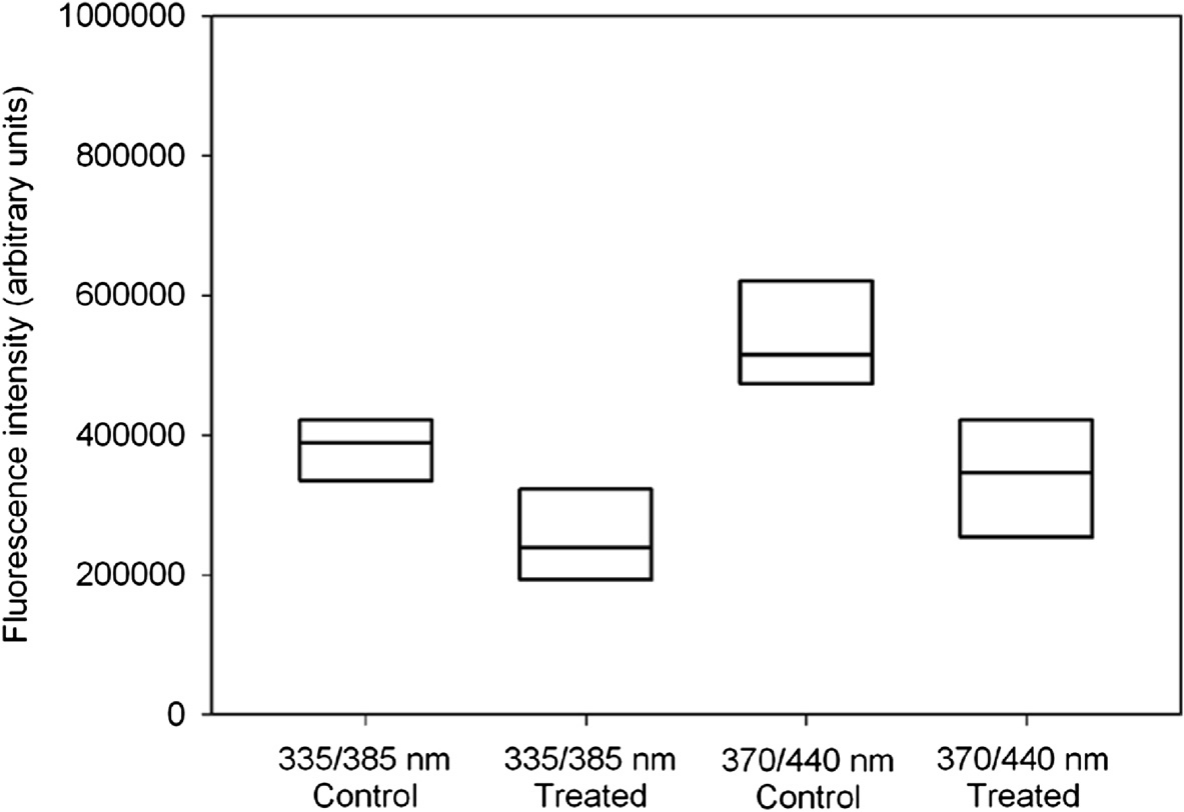
**Box plot (lower 25 percentile, median and upper 75 percentile) of fluorescence measurements in control lenses and lenses treated with photobleaching.** Fluorescence was measured for argpyrimidine and pentosidine-like AGEs (λ_em_/λ_ex_ 335/385 nm) and for other AGEs (λ_em_/λ_ex_ 370/440 nm).

In addition, we measured the fluorescence intensity of argpyrimidine and pentosidine-like AGEs as the emission peak at 385 nm after excitation at 335 nm and the fluorescence intensity of other AGEs was measured at the fluorescence peak at 440 nm after excitation at 370 nm in 7 lens pairs, see Figure 4. Fluorescence was on average 25.9% ± 22.3% (mean ± SD) lower after photobleaching at λ_em_/λ_ex_ 335/385 nm and it was 32.3% ± 20.8% (mean ± SD) lower at λ_em_/λ_ex_ 370/440 nm. The difference was significant for both λ_em_/λ_ex_ 370/440 nm and λ_em_/λ_ex_ 335/385 nm (p = 0.02, signed rank sum test).

## Discussion

Cataract is an important health care problem. We have proposed to treat cataracts non-invasively by photobleaching the lens. The aim of the present study was to examine the effect of the photobleaching on the optical properties of the lens and to characterize changes in one set of posttranslational protein modifications responsible for cataract formation, advanced glycation end products. We found that the photobleaching procedure resulted in increased transmission of visible light and decreased lens autofluorescence indicating that the intrinsic lens chromophores and fluorophores were altered by the procedure. We did not find an effect on the scattering properties, probably because all treated lenses showed very little scattering prior to treatment and hence no improvement could be expected.

The visual disturbance experienced by patients with cataract is related to absorption and scattering of visible light. We found that transmission of light increased after treatment with the greatest increase in the blue part of the visible spectrum. This shows that blue-light absorbing chromophores were bleached by the photobleaching procedure. Absorption of blue light is a dominant feature of lens aging [19]. Thus, the photobleaching procedure may have the potential to improve visual function in elderly patients with strongly absorbent lenses but the exact effects on visual function can only be evaluated by clinical testing. We were, however, not able to determine which chromophores were photobleached by the procedure in the present study. The ageing human lens contains a wide range of chromophores. The tryptophan metabolites, such as the kynurenines and xanthurenic acid, all absorb light in UVA-range [24]. Most advanced glycation end products, such as vesperlysine [25], K2P [26] and OP-lysine [27] also absorb light in the UVA range. To the best of our knowledge, no chromophores absorbing with a peak around 440–450 nm has yet been described. Thus, the chromophores responsible for the effect observed in the present study remain to be discovered. The nature of the photochemical reactions involved in the photobleaching also remains to be further explored.

Irradiation of lenses with short wavelength light is usually thought of as cataractogenic. Irradiation with ultraviolet radiation, especially UVB, has been shown to lead to cataract formation in several animal species [28-30]. However, irradiation with longer wavelength UVA at 370 nm did not result in any observable damage to porcine lenses whereas the threshold for cataract formation was very low when shorter wavelengths of UV, i.e. 280–290 nm, was used [31]. In the present study we used irradiation at 445 nm and did not find any observable harmful effects. Due to difficulties preserving human lenses for a prolonged time period in the laboratory and because lens samples were taken immediately after the photobleaching procedure for analysis of advanced glycation end products, the observations on potential harmful effects are limited to the immediate post-treatment period.

## Conclusions

In conclusion, we found that transmission of visible light increased significantly after photobleaching in human lenses and that the AGE-like fluorescence also decreased after photobleaching, but these optical changes were not accompanied by a decrease in the concentration of the measured advanced glycation end products. However, we like to point out that we have measured only a few advanced glycation products, of which two (carboxymethyllysine and hydroimidazolone) are non-chromophoric and the decreased transmission in AGE-like fluorescence would have had to be caused by other AGEs than those we measured directly. Thus, the products we measured should be treated only as markers for advanced glycation end products. There could be many more advanced glycation products that could be affected by our photobleaching treatment; further studies are needed to verify this possibility. Thus, the chromophores involved in photobleaching remains to be elucidated.
